# Comparative Evaluation of the Remineralizing Potential of Fluoridated and Non-fluoridated Agents on Demineralized Primary Tooth Enamel: An In Vitro Study

**DOI:** 10.7759/cureus.85732

**Published:** 2025-06-10

**Authors:** Anushka Gayan, Ashish Sinha, Seema Chaudhary, Bornisha Bezborah, Somy Agarwal, Shilpi Kumari, Niharika Sharma

**Affiliations:** 1 Department of Paediatric and Preventive Dentistry, Kothiwal Dental College and Research Centre, Moradabad, IND; 2 Department of Paediatric and Preventive Dentistry, Grace Dental Clinic, Guwahati, IND; 3 Department of Paediatric and Preventive Dentistry, Shreya Hospital, Ghaziabad, IND; 4 Department of Paediatric and Preventive Dentistry, Mithila Minority Dental College and Hospital, Darbhanga, IND; 5 Department of Paediatric and Preventive Dentistry, Institute of Dental Sciences, Jammu, IND

**Keywords:** demineralization, dental caries, hardness, primary teeth, remineralization, surface

## Abstract

Introduction: Dental caries remains a highly prevalent condition in children, with primary teeth being particularly susceptible due to their lower mineral content and structural characteristics. Early intervention using remineralizing agents can reverse enamel demineralization and prevent disease progression. The aim of this study was to evaluate and compare the remineralizing efficacy of various fluoridated and non-fluoridated agents on the surface microhardness (SMH) of demineralized primary tooth enamel.

Materials and methods: This in vitro experimental study was conducted on 50 extracted, caries-free primary molars, randomly divided into five groups as Group 1, who received casein phosphopeptide-amorphous calcium phosphate (CPP-ACP) paste (GC Tooth Mousse®, GC Corporation, Tokyo, Japan) (n = 10); Group 2, who was treated with nano-hydroxyapatite (nano-HA) toothpaste (Perfora™, Perfora Oral Care Pvt. Ltd., India) (n = 10); Group 3, who received grape seed extract solution (*Vitis vinifera*, Biomed Sensitive Toothpaste, Splat Global LLC, Moscow, Russia) (n = 10); Group 4, who was treated with sodium fluoride (NaF) (Mamaearth Natural Toothpaste, Honasa Consumer Pvt. Ltd., Haryana, India) (n = 10) serving as the positive control with 0.165% w/w NaF, which is equivalent to approximately 750 parts per million (ppm) fluoride; and Group 5, who was treated with normal saline (Baxter Healthcare Corporation, Deerfield, IL) (n = 10) serving as the negative control. Artificial enamel lesions were created using demineralizing solution (pH 4.5) for 72 hours. The agents were applied for four minutes daily for seven days. The samples were stored in artificial saliva for various applications. The SMH was measured using a Vickers microhardness tester at three time points: baseline (T0), after demineralization (T1), and after remineralization (T2). Statistical analysis was performed using one-way analysis of variance (ANOVA), paired t-tests, and Tukey’s post-hoc tests (p < 0.05).

Results: Post-remineralization analysis revealed statistically significant differences between groups (p = 0.002). The paired t-test showed that Groups 1 and 4 exhibited the most significant microhardness improvement. Group 2 showed moderate efficacy, whereas Groups 3 and 5 showed no significant remineralization effects. Post-hoc analysis confirmed the superior performance of Groups 1 and 4 compared to Group 5.

Conclusion: CPP-ACP and NaF demonstrated superior remineralization potential in demineralized primary enamel. Nano-HA showed moderate efficacy, whereas grape seed extract and saline were ineffective. These findings support the use of fluoridated and non-fluoridated agents in pediatric preventive dentistry.

## Introduction

Dental caries remains one of the most widespread and chronic oral health conditions affecting individuals of all ages, particularly in children [1]. According to Pandey et al. [2], the prevalence of dental caries in India was 54.16%. Dental caries significantly impairs not only oral function but also overall quality of life by causing pain, discomfort, and difficulty in eating and speaking and affects the overall mental health of patients [3]. Despite advances in oral health care, dental caries continues to be a global public health issue, particularly in children, often termed as a “silent pandemic” due to its high prevalence and large number of untreated cases [4].

The caries process begins when acidic by-products of bacterial metabolism, primarily dietary carbohydrates, lower the pH of the oral cavity. Once the pH drops below 5.5, enamel, which is the highly mineralized outer layer of the tooth, begins to lose minerals such as calcium and phosphate, leading to a stage known as white spot lesion [5]. This marks the early onset of enamel demineralization. Fortunately, this process is reversible in its early stages through remineralization, a natural repair mechanism supported by saliva. However, the capacity of saliva alone to restore lost minerals is limited, especially in cases of frequent acid attacks or compromised salivary flow [6].

Children are particularly more susceptible to caries than adults due to several factors. Primary teeth differ structurally from permanent teeth as they have a higher organic content, thinner enamel, and lower mineral density, making them more vulnerable to acid attack [7]. Moreover, the dietary habits of children, often rich in sugars and processed foods, further increase their risk for rapid caries progression, known as early childhood caries (ECC) [8]. Addressing this issue requires minimally invasive preventive strategies that preserve tooth structure and enhance resistance to decay.

One such strategy is the application of remineralizing agents, which are substances designed to deliver calcium, phosphate, and sometimes fluoride ions directly to demineralized enamel surfaces [5]. Fluoride has long been recognized as a cornerstone of caries prevention. It enhances remineralization, inhibits demineralization, and interferes with bacterial metabolism. Fluoride ions help form fluorapatite, a crystal more resistant to acid than natural hydroxyapatite, offering increased protection against future acid attacks [9].

In recent years, non-fluoridated alternatives have gained attention. Casein phosphopeptide-amorphous calcium phosphate (CPP-ACP) uses milk-derived proteins to stabilize and deliver calcium and phosphate ions to enamel [10]. Nano-hydroxyapatite, which closely resembles the natural mineral component of enamel, promotes remineralization by directly integrating into the enamel structure [11]. Grape seed extract (GSE), a natural product rich in proanthocyanidins, supports remineralization by strengthening collagen and forming mineral-rich deposits on enamel surfaces [12].

Given the variety of available agents, both fluoridated and non-fluoridated, there is a growing need to evaluate and compare their effectiveness, particularly in the context of the primary teeth. The present in vitro study aimed to assess and compare the effects of different remineralizing agents on the surface microhardness of demineralized primary tooth enamel, offering valuable insights into their potential role in pediatric preventive dentistry. The null hypothesis (H₀) of this study stated that there would be no significant difference in the remineralizing potential of various fluoridated and non-fluoridated agents on the surface microhardness of demineralized primary tooth enamel.

## Materials and methods

Study design

The present in vitro experimental study was carried out in the Department of Pediatric and Preventive Dentistry at the Kothiwal Dental College and Research Centre, Moradabad, India, from June 2022 to January 2023. Ethical approval (KDCRC/IERB/02/2022/30) was obtained before starting the study, and written informed consent was obtained from all patients to use their extracted teeth for study purposes. The study followed the principles of the Declaration of Helsinki.

Sample size calculation

The sample size was calculated using G*Power software (version 3.1.9.3; Franz Faul, University of Kiel, Germany) based on a priori analysis of variance (ANOVA) fixed-effect omnibus analysis (F-test) with an effect size of 0.58 considered from a previous study [13], an alpha error probability of 0.05, and a power of 0.80. This yielded the required sample size of 45 specimens (nine per group), which was increased to 50.

Methodology

Fifty intact human first and second primary molars (age 5-10 years) were selected for the study. The teeth were cleaned of calculus, soft tissue, and debris with hand instrumentation and stored in saline for a maximum of one month after extraction. The saline solution was changed every two days. Changing saline every two days prevents bacterial growth, maintains tissue integrity, and ensures hydration stability for accurate stereomicroscopic examination. All tooth surfaces were examined thoroughly using a stereomicroscope (SZ 61; Olympus, Tokyo, Japan) at 10x magnification.

The study included extracted primary molars with intact enamel, free from cracks, hypoplasia, caries, or prior restorative treatments. Only non-fluorotic teeth subjected to standardized artificial demineralization were selected. Exclusion criteria comprised teeth with structural defects, fluorosis, caries, dehydration, or prior fluoride exposure. Additionally, teeth exhibiting excessive wear were excluded. Samples lost during processing or lacking complete data were also discarded.

The collected teeth were randomly divided into five groups. Group 1 received CPP-ACP paste (GC Tooth Mousse®; GC Corporation, Tokyo, Japan) (n = 10); Group 2 was treated with nano-hydroxyapatite toothpaste (Perfora™; Perfora Oral Care Pvt. Ltd., India) (n = 10); Group 3 received grape seed extract solution (*Vitis vinifera*, Biomed Sensitive Toothpaste; Splat Global LLC, Moscow, Russia) (n = 10); Group 4 was treated with sodium fluoride (Mamaearth Natural Toothpaste; Honasa Consumer Pvt. Ltd., Haryana, India) (n = 10) serving as the positive control with 0.165% w/w sodium fluoride, which is equivalent to approximately 750 parts per million (ppm) fluoride; and Group 5 was treated with normal saline (Baxter Healthcare Corporation, Deerfield, IL) (n = 10) serving as the negative control.

The samples were disinfected and cleaned using an ultrasonic scaler (Woodpecker®, Guilin Woodpecker Medical Instrument Co., Ltd., China). The crowns were separated from the roots at the cementoenamel junction using a diamond cutting disc (NTI Diamond Disc, Kerr Dental, Orange, CA) attached to a low-speed micromotor and a straight handpiece (NSK®; Nakanishi Inc., Japan). The sectioned crowns were embedded in autopolymerizing acrylic resin (DPI-RR Cold Cure®; Dental Products of India Ltd., Mumbai, India) using cylindrical molds. The buccal enamel surfaces were polished with silicon carbide abrasive papers (3M® Wetordry™; 600 and 1200 grit, Saint Paul, MN) to obtain a smooth and standardized surface for testing.

**Figure 1:**
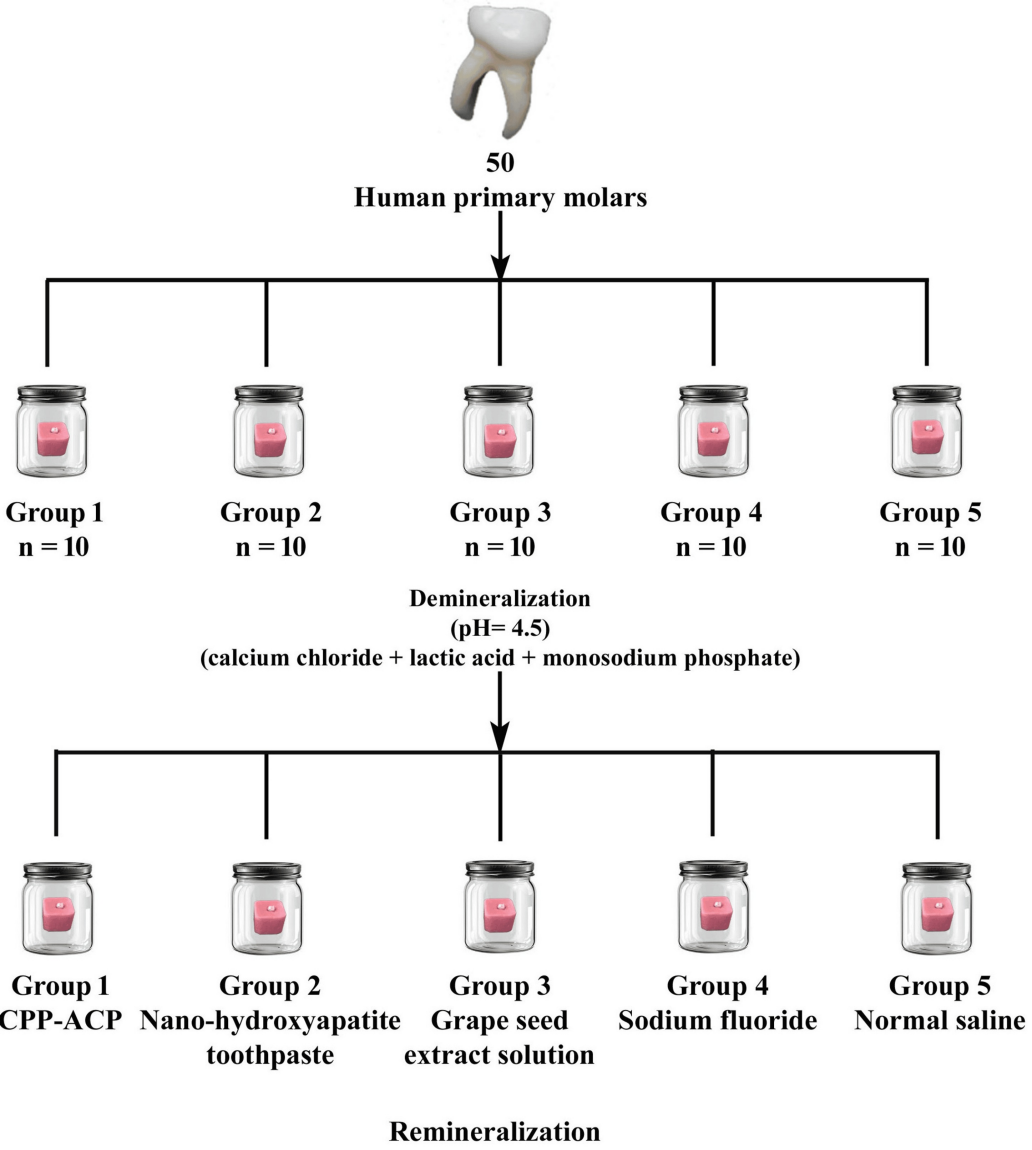
Demineralization and remineralization of the tooth surface. n = number of samples in group. Group 1 received casein phosphopeptide-amorphous calcium phosphate (CPP-ACP) paste; Group 2 was treated with nano-hydroxyapatite toothpaste; Group 3 received grape seed extract solution; Group 4 was treated with sodium fluoride; and Group 5 was treated with normal saline. This image is created by the author.

To create artificial carious lesions, the specimens were immersed for 72 hours in a 50 mL demineralizing solution at 37 °C in an incubator (Coslab, ISO 9001:2000; Ambala Cant, Haryana, India). The demineralizing solution was prepared using 2.2 mM calcium chloride (Loba Chemie Pvt. Ltd., Mumbai, India), 2.2 mM monosodium phosphate (Loba Chemie Pvt. Ltd., Mumbai, India), and 0.05 M lactic acid (Loba Chemie Pvt. Ltd., Mumbai, India), with the pH adjusted to 4.5 using 50% sodium hydroxide (Loba Chemie Pvt. Ltd., Mumbai, India) [13]. After the demineralization phase, the teeth were rinsed with deionized water and air-dried. Remineralization was performed by applying the respective agents for four minutes every 24 hours over a seven-day period. The application was performed using microbrush applicators (Dentsply Sirona®, Charlotte, NC), followed by rinsing with deionized water (Figure 1).

The samples were stored in an incubator with artificial saliva, which was replaced daily [14]. The surface microhardness (SMH) of the enamel was assessed using a Vickers microhardness testing machine (FM-800; Future-Tech Corp., Kawasaki, Japan). A load of 200 g was applied for 15 seconds using a diamond indenter, and three indentations were made on each sample. The mean values of the three readings were calculated and used for the analysis. To ensure accuracy and reliability, all the microhardness tests were performed using a single calibrated operator. Intra-examiner reliability was verified by re-evaluating 10% of the samples after one week and computing the intraclass correlation coefficient (ICC), which provided a value of 0.92, showing excellent reliability. SMH was recorded at baseline (T0), after demineralization (T1), and after remineralization (T2) in all groups.

Statistical analysis

Statistical analysis was performed using the Statistical Product and Service Solutions (SPSS, version 26; IBM SPSS Statistics for Windows, Armonk, NY). The normality of the data was tested using Shapiro-Wilk’s test and found to be normally distributed (p > 0.05). Therefore, the significance of the mean difference was tested using parametric tests. The mean microhardness between the groups was tested using one-way ANOVA, followed by post-hoc Tukey’s test. The mean microhardness was also compared intragroup between two different time points using a paired t-test. The level of significance and confidence interval were set at 5% and 95%, respectively.

## Results

Paired t-test analysis demonstrated that remineralization outcomes varied notably across the five groups. Groups 1 and 4 showed the most effective remineralization, as evidenced by substantial increases in microhardness and large effect sizes, indicating a strong treatment impact. Group 2 experienced moderate but statistically significant improvement, suggesting partial efficacy. Conversely, Groups 3 and 5 did not exhibit significant changes in microhardness, reflecting the limited or no remineralizing potential of the agents used in these groups. Overall, the findings highlighted different remineralizing capacities, with Groups 1 and 4 being the most effective (Table 1).

**Table 1:** Mean surface microhardness (SMH) comparison at different time points by paired t-test. SMH has been represented in the form of mean ± standard deviation (SD). *p-value < 0.05: significant Group 1: CPP-ACP paste, Group 2: nano-hydroxyapatite toothpaste, Group 3: grape seed extract solution, Group 4: sodium fluoride as the positive control, Group 5: normal saline as the negative control.

Groups	n (%)	Demineralization	Remineralization	t value	p-value	Effect size
		Mean ± SD	Mean ± SD			
Group 1	10 (20%)	61.8 ± 5.37	77.98 ± 6.64	6.00	0.001*	2.68
Group 2	10 (20%)	62.69 ± 9.87	75.43 ± 12.29	2.56	0.021*	1.14
Group 3	10 (20%)	68.87 ± 8.27	73.47 ± 9.07	1.18	0.251	0.53
Group 4	10 (20%)	59.3 ± 7.21	81.68 ± 9.01	6.14	0.001*	2.74
Group 5	10 (20%)	64.26 ± 4.55	65.15 ± 4.73	0.43	0.673	0.19

One-way ANOVA assessed the mean SMH differences between the five groups after demineralization and revealed no statistically significant variation (p = 0.067). The effect size (0.21) was small, implying that group differences had minimal practical significance. This suggested that the demineralization process resulted in comparable SMH across groups, with no single group exhibiting distinctly different SMH values post-demineralization, supporting homogeneity in baseline conditions (Table 2).

**Table 2:** One-way analysis of variance (ANOVA) between groups after demineralization. df: degree of freedom, p-value > 0.05: non-significant

Variable	Sum of Squares	df	Mean Square	F value	p-value	Effect size
Group	505.33	4	126.33	2.36	0.067	0.21
Residual	2406.51	45	53.48			

The null hypothesis was rejected as one-way ANOVA analysis showed a statistically significant variation (p = 0.002) between the groups after remineralization. The significant p-value confirmed that at least one group differed significantly in SMH post-remineralization. The moderate effect size (0.45) indicated practical significance, suggesting that remineralization treatments had varying efficacy across groups. This implied that certain groups achieved greater microhardness improvements, highlighting the influence of different remineralization protocols (Table 3).

**Table 3:** One-way analysis of variance (ANOVA) between groups after remineralization. df: degree of freedom, *p-value < 0.05: significant

Variable	Sum of Squares	df	Mean Square	F value	p-value	Effect size
Group	1527.18	4	381.8	5.01	0.002*	0.45
Residual	3426.28	45	76.14			

Post-hoc Tukey's analysis identified significant differences in the remineralization efficacy between specific groups. Both Groups 1 and 4 achieved significantly higher microhardness values than Group 5, underscoring the superior performance of their respective agents. The absence of significant differences among Groups 1, 2, 3, and 4 suggested comparable remineralization outcomes within this subset. However, Group 5 consistently demonstrated inferior results, reinforcing its limited effectiveness in enhancing enamel microhardness after demineralization (Table 4).

**Table 4:** Pairwise comparison of groups using the post-hoc Tukey's test. *p-value < 0.05: significant, CI: confidence interval, Group 1: CPP-ACP paste, Group 2: nano-hydroxyapatite toothpaste, Group 3: grape seed extract solution, Group 4: sodium fluoride as the positive control, Group 5: normal saline as the negative control

Pair-Wise Groups	Mean Difference	p-value	95% CI Lower Limit	95% CI Upper Limit
Group 1 - Group 2	2.55	0.965	-8.54	13.64
Group 1 - Group 3	4.51	0.776	-6.58	15.6
Group 1 - Group 4	3.7	0.876	-7.39	14.79
Group 1 - Group 5	12.83	0.016*	1.74	23.92
Group 2 - Group 3	1.96	0.987	-9.13	13.05
Group 2 - Group 4	6.25	0.504	-4.84	17.34
Group 2 - Group 5	10.28	0.081	-0.81	21.37
Group 3 - Group 4	8.21	0.236	-2.88	19.3
Group 3 - Group 5	8.32	0.225	-2.77	19.41
Group 4 - Group 5	16.53	0.001*	5.44	27.62

## Discussion

The findings of this in vitro study provide valuable insights into the comparative efficacy of various remineralizing agents on the SMH of artificially demineralized enamel in primary molars. This study evaluated five groups treated with CPP-ACP paste, nano-hydroxyapatite toothpaste, GSE solution, sodium fluoride (positive control), and normal saline (negative control). The results demonstrated significant differences in the remineralization outcomes.

The superior performance of CPP-ACP can be attributed to its multifaceted mechanism of action, which makes it a highly effective remineralizing agent. CPP-ACP delivers bioavailable calcium and phosphate ions through stabilized ACP complexes, significantly enhancing enamel remineralization by surpassing the concentrations of free calcium and phosphate ions found in saliva [15]. The cluster sequence of Ser(P)-Ser(P)-Glu-Glu in CPP increases the apparent solubility of calcium phosphate by stabilizing ACP, particularly under neutral to alkaline conditions, while maintaining efficacy at acidic pH levels [16]. This facilitates robust mineral deposition into the enamel lattice, effectively reversing early lesions, particularly in primary teeth with thinner enamel. Beyond remineralization, CPP-ACP resists plaque accumulation by binding to the pellicle, substituting for albumin, and inhibiting the adhesion of cariogenic bacteria, such as *Streptococcus mutans* and *Streptococcus pneumoniae*, thus promoting non-cariogenic plaque formation [17]. Additionally, the high water solubility of CPP-ACP ensures the efficient delivery of ions, and its buffering capacity may contribute to preventing root caries [17]. Its versatility extends to alleviating dentin hypersensitivity by occluding dentinal tubules. These combined properties, remineralization, anti-cariogenic effects, and hypersensitivity relief, underscore the superior performance of CPP-ACP compared to other agents tested, making it a highly promising option for clinical applications in pediatric dentistry. Our findings were supported by previous studies [18,19], where CCP-ACP was found to be superior to fluoride in remineralizing early carious lesions.

Sodium fluoride, used as the positive control, also demonstrated significant remineralization, which is consistent with its well-established role in caries prevention and enamel repair. Fluoride ions enhance remineralization by forming fluorapatite, which is a more acid-resistant mineral phase, within the enamel structure. The protective action of fluoride involves the formation of a calcium fluoride (CaF2)-like layer on the tooth surface, which facilitates the remineralization process by encouraging the reconstruction of minerals such as fluorapatite or fluorohydroxyapatite on the enamel crystal surfaces [9]. This finding aligns with a previous research, where the effect of CPP-ACP versus fluoride toothpaste was evaluated, and it was concluded that toothpaste containing fluoride at concentrations of 500 ppm or higher demonstrated superior inhibition of lesion progression compared to toothpaste containing CPP or 260 ppm fluoride concentration [20]. Similarly, Oliveira et al. [21] reported that 1.1% sodium fluoride at 5,000 ppm showed better results than CPP-ACP, likely because of the enhanced mineral reconstruction facilitated by fluoride. However, contrasting results were reported in another study, where CPP-ACP’s effectiveness in reducing lesion depth surpassed that of fluoride toothpaste, attributed to ultrafine ACP nanocomplexes that enable deep penetration of phosphate and calcium ions into porous lesions [22]. Similarly, a separate study demonstrated that enamel surfaces treated with CPP-ACP paste exhibited superior remineralizing potential compared to fluoride toothpaste [23]. Despite these discrepancies, the results of the present study reinforce the robust efficacy of sodium fluoride, particularly at 750 ppm, in enhancing enamel microhardness, suggesting its continued relevance as a gold standard in remineralization therapies. The comparable efficacy of CPP-ACP and sodium fluoride in this study indicated that both agents were highly effective and potentially offered complementary benefits in clinical settings, with fluoride providing superior acid resistance and CPP-ACP delivering bioavailable ions for deeper lesion repair.

Nano-hydroxyapatite showed moderate but statistically significant improvements in SMH, indicating partial efficacy. Nano-hydroxyapatite mimics the natural composition of enamel, releasing calcium and phosphate ions that integrate into the demineralized enamel matrix. Its small particle size enhances penetration and adhesion to the enamel surface, facilitating remineralization [24]. However, its performance was less pronounced than that of CPP-ACP and sodium fluoride, possibly because of differences in ion release kinetics or interaction with the enamel surface under the experimental conditions. Similar results were obtained by Rane et al. [25], who reported the highest remineralization with CCP-ACP, followed by sodium fluoride and nano-hydroxyapatite.

GSE showed an increase in SMH, but less than CPP-ACP and nano-hydroxyapatite, with the lowest mean SMH value as compared to other remineralizing agents. GSE contains proanthocyanidins (PA), which are hypothesized to stabilize collagen in the enamel matrix and inhibit bacterial adhesion, potentially indirectly aiding remineralization. The discrepancy in its performance may be attributed to the higher molecular weight of PA molecules, which potentially limits their penetration into the underlying layers of enamel [26]. Consequently, PA primarily facilitated mineral deposition on the surface of the lesion, inhibiting further mineral deposition in the deeper parts of the lesion, as supported by a previous study [27]. Contrary to the present study, Desai et al. [28] investigated the efficacy of remineralization between GSE and CPP-ACP and found that GSE showed better results than CPP-ACP, which was attributed to its ability to induce alterations within the organic matrix through the emergence of freshly induced collagen cross-linkages. The limited efficacy observed in this study might be due to the short application period (seven days) or the specific concentration used, suggesting that further research is needed to optimize formulations, application durations, or synergistic effects with other agents to enhance their remineralizing potential.

As expected, normal saline showed no significant remineralizing effect and served as an effective negative control. The absence of active remineralizing agents in this group highlighted the necessity for therapeutic interventions to reverse demineralization, as spontaneous remineralization in the absence of calcium, phosphate, or fluoride ions was minimal. The significant differences between Group 5 and Groups 1 and 4 in the post-hoc analysis reinforce the effectiveness of CPP-ACP and sodium fluoride in enhancing enamel microhardness. The homogeneity in SMH post-demineralization across all groups, as indicated by the non-significant one-way ANOVA results (p = 0.067), ensured that the baseline conditions were comparable, strengthening the validity of the remineralization comparisons. The significant variation in SMH post-remineralization (p = 0.002) and moderate effect size (0.45) highlighted the differential efficacy of the tested agents, underscoring the importance of selecting appropriate remineralizing agents based on their mechanisms and clinical performance.

A standardized demineralization-remineralization protocol provided a robust framework for evaluating enamel changes. High intra-examiner reliability (ICC = 0.92) enhanced the reliability of the SMH measurements, ensuring that the observed differences were attributable to the treatments rather than operator variability. The use of artificial saliva mimicked the oral environment and improved the clinical relevance of the findings, although it did not fully replicate the dynamic conditions of the oral cavity.

Clinical implications

These findings have significant implications for pediatric dentistry, particularly in the management of early enamel lesions in primary teeth. CPP-ACP and sodium fluoride have emerged as highly effective remineralizing agents suitable for incorporation into preventive and therapeutic protocols. CPP-ACP can be recommended for home or in-office applications, especially for young children who may benefit from its non-fluoride-based mechanism, reducing the risk of fluorosis in developing teeth. Sodium fluoride, widely available in varnishes and gels, remains the cornerstone of caries prevention and should continue to be a primary choice in clinical practice. Nano-hydroxyapatite toothpaste offers a viable alternative, particularly for patients seeking fluoride-free options or for those with specific sensitivities. Its moderate efficacy suggests that it could be used as part of a comprehensive preventive regimen, potentially in combination with other agents, to enhance outcomes. The limited efficacy of GSE indicates that it may not be a primary choice for remineralization, but could be explored as an adjunctive agent in future research, possibly due to its antibacterial or collagen-stabilizing properties.

Clinicians should consider patient-specific factors such as compliance, age, and risk of caries when selecting remineralizing agents. The application protocol used in this study (four minutes daily for seven days) provides a practical framework for clinical use, although long-term studies are needed to assess its sustained effects. Preventive strategies incorporating these agents can reduce the progression of early carious lesions, minimize the need for invasive interventions, and promote long-term oral health in pediatric patients.

Limitations

Despite its strengths, this study has several limitations. First, the in vitro design limits clinical generalizability, as it cannot fully replicate the oral environment’s complex factors, including saliva flow, microbial activity, and dietary influences. Second, the seven-day remineralization period may not reflect long-term outcomes, as remineralization is a gradual process requiring extended exposure for maximal effects. Third, the study relied solely on SMH as an outcome measure, which, while reliable, does not capture other aspects of enamel integrity, such as mineral content or structural changes, assessable via techniques such as scanning electron microscopy or X-ray diffraction. Furthermore, despite being based on statistical power calculations, the small sample size (n = 10 per group) may limit the detection of subtle group differences. Finally, the concentration and application method of GSE may not have been optimized, potentially underestimating its efficacy. Future studies should address these limitations by incorporating in vivo models, longer treatment durations, and additional outcome measures to provide a more comprehensive evaluation of remineralizing agents, such as GSE, CPP-ACP, or fluoride.

## Conclusions

This in vitro study demonstrated that sodium fluoride and CPP-ACP were the most effective remineralizing agents for enhancing the SMH of artificially demineralized enamel in primary molars, with sodium fluoride exhibiting the highest SMH among all the groups. Nano-hydroxyapatite showed moderate efficacy, whereas GSE and normal saline exhibited limited or no significant remineralizing potential. The superior performance of sodium fluoride and CPP-ACP highlights their potential as primary therapeutic options for managing early enamel lesions in pediatric dentistry. These findings underscore the importance of selecting remineralizing agents based on their mechanisms and clinical efficacy; however, further in vivo studies with longer durations and additional outcome measures are warranted to validate these results in clinical settings.
